# Risks and Benefits of Dual Antiplatelet Therapy Beyond 12 Months After Coronary Stenting: A Prospective Randomized Cohort Study

**DOI:** 10.1097/MD.0000000000003663

**Published:** 2016-06-03

**Authors:** Yahya Dadjou, Salar Safavi, Javad Kojuri

**Affiliations:** From the Atherosclerosis Research Center, Baqiyatallah University of Medical Science, Tehran (YD); Quality Improvement in Clinical Education Research Center, Shiraz University of Medical Sciences (SS); and Quality Improvement in Clinical Education Research Center, Shiraz Cardiovascular Research Center, Zand St Namazi Hosp. Cardiology Office (JK), Shiraz, Iran.

## Abstract

The optimal duration of dual antiplatelet therapy (DAT) after coronary stenting remains poorly define. The aim of this study was to evaluate the impact of longer than 24 months DAT in patients who received drug-eluting and bare-metal stents.

A total of 1010 individuals who underwent elective, urgent or emergency coronary angioplasty with intended stent implantation at reference or specialized cardiac hospitals were randomized to receive long-term and short-term DAT to determine the benefits and adverse effects of long-term DAT. Total of 508 patients were randomized to long-term and 502 patients to <1 year DAT, and all of them were followed for more than 36 months for major adverse cardiac and cerebvascular events and bleeding major adverse cardiac and cerebvascular events (MACCE)

Mean age of the 1010 patients (364 women and 646 men) was 60 years. Stent reocclusion occurred in 15 patients. Mean Syntax score was 23.00 ± 5.08 for whole samples, 25.00 ± 5.27 in 28 patients with MACCE and 23 ± 5.00 in 982 patients without MACCE (*P* = 0.057). According to all specified bleeding definitions, clopidogrel therapy for >12 months was not associated with a greater risk of hemorrhage. A regimen of >12 months of clopidogrel therapy in patients who had received drug-eluting or bare-metal stents did not differ significantly from a regimen of <12 months on clopidogrel with regard to MACCE.

Long-term DAT might not significantly affect the reduction in the risk of death from any cause, myocardial infarction, or stroke, and not associated with minor or major bleeding events.

## INTRODUCTION

It is predominantly anticipated by previous studies that clopidogrel therapy for a year after bare-metal stenting appears to be effective in death rate reduction, myocardial infarction, and stroke by nearly about 30% in patients with non-ST-segment-elevation acute coronary syndrome.^1,2^ Long-term clopidogrel therapy has been also recommended to patients who suffer from acute coronary syndromes, as well as patients who have undergone coronary stenting.^3,4^

Numerous studies have been carried out to evaluate the impact clopidogrel therapy on patients’ recovery; according to the results derived from mentioned studies and due to American College of Cardiology/American Heart Association (AHA) guidelines and European Society of Cardiology clopidogrel therapy should last for at least a year after drug-eluting stent implantation procedure.^5,6^ The mentioned advice is based on the reasons that delayed vessel healing which may be responsible for late (>30 days) or very late (>1 year) stent thrombosis. Actually, there is not enough information about different aspects of mentioned guidelines and recommendations,^7^ it is also important to state that observational studies’ findings have been inconsistent.^8–11^ This will increase the dilemma when different stent scaffold, polymers, and active drugs come in to consideration. Therefore, the optimal dual type of antiplatelet therapy duration and the risk–benefit ratio for long-term type of dual antiplatelet therapy (DAT) after percutaneous coronary interventions (PCIs) still remains uncertain. There is only 1 open-label clinical trial conducted for prolonged DAT at 2 centers which was designed to assess the efficacy and safety evaluation of prolonging clopidogrel therapy for up to 2 years in all referred patients who have received a balanced combination of stents.^12,13^

To date, the effect of long-term type of clopidogrel therapy (especially longer than a year) is unknown. This study was conducted under the goal of determining the effect of long-term DAT (>1 year) on clinical outcomes (major cardiovascular events and bleeding) after coronary intervention in a broad all-comers patient population who received a balanced proportion of different Food and Drug Administration-approved stents.

## METHODS

In this study, 1010 participants, consisting of all patients who were referred for elective, urgent, or emergency coronary angioplasty with intended stent implantation at reference or specialized cardiac hospitals, were selected randomly to be enrolled in the study to receive longer than a year or less, DAT for the years 2011 to 2012 based on simple randomization. Totally 1010 patients received a different type of stent according to operator choice (e.g., Xience, Promus, Apolo, Biomarix, Clearflex, Cypher, Endovear, Infinium, Multi link, etc.). Random allocation was done to divide patients into 2 groups by specialized personnel blinded to results and all data gathered in follow-up and analysis, and data analysis also was done by researchers blinded to group of patients. The inclusion criteria were broad and any patient with stenosis more than 70% in any coronary vessel with reference diameter of more than 2.25 which was suitable for coronary stenting, was considered as potential case. There was no limitation in the number of stented lesions, vessels, or, length of stenosis. Exclusion criteria were allergy to aspirin or clopidogrel, planned surgery within 6 months of PCI unless the DAT could be continued throughout the perioperation period, history of bleeding diathesis, major surgery within 15 days, active bleeding, previous hemorrhagic stroke in the past 6 months which contraindicated use of DAT, pregnancy, life expectancy <24 months, and inability to provide informed consent. No patient was excluded from the study, based on comorbid conditions or, age.

According to protocol, all patients received aspirin (325 mg chewing as a loading dose, then 240 mg for 2 months, and then 75 mg/d lifelong) and clopidogrel (600 mg orally as a loading dose, then 75 mg daily for the duration of treatment).

All interventions were performed based on the last guidelines, and the final interventional strategy, including administration of glycoprotein IIb/IIIa antagonist, predilation, or postdilation, or the use of intravascular imaging, depended on operator's decision.

Patients who have not missed the follow-up sessions, regardless of their compliance with the assigned treatment schedule, returned for study visits after 30 days and then every 6 months up to the end of the follow-up period. During follow-up sessions, occurrence of 2 groups of minor and major adverse effects were examined and assessed among patients; for example, intracranial and gastrointestinal hemorrhage, need for hospitalization because of bleeding, severe bleeding from the surgical wound, nasal bleeding, hematuria, skin rash, hemoptysis, or gum bleeding. At each follow-up session, all patients were asked whether they had compliance with the medication used in study or not. Any interruption or termination of treatment as well as the factors affecting the situation were recorded. To ensure a high rate of adherence to the assigned study treatment, 2 dedicated and educated nurses were in touch with each patient monthly using communicative device such as phones and cell phones.

Numerous significant risk factors and predisposing disorders such as smoking, chronic kidney disease, diabetes mellitus, and gastrointestinal disorders have been considered in the present study. Due to Academic Research Consortium criteria, secondary end points including each component of the primary end point, cardiovascular death, the incidence of stent reocclusion and bleeding outcomes were defined.^14^ The key safety end point was the rate of bleeding due to TIMI^1^ criteria and the Bleeding Score.^15^ Prespecified analysis of the primary and secondary end points was done based on age, gender, presence of diabetes mellitus, type of stent, smoking, clinical presentations, complexity of lesions, number of treated lesions, and kidney function. All reported death and their causes were recorded and classified as cardiovascular or noncardiovascular types. The acute myocardial infarction was diagnosed based on the universal definition.^16^ Stroke and transient ischemic attacks were diagnosed by the use of brain computed tomography scan and magnetic resonance imaging (as needed) and confirmation of neurologist. The composite of death, myocardial infarction, cerebrovascular accident, and stent restenosis or thrombosis, proved by angiography, were considered MACCE and as secondary endpoint, followed by trained personnel by direct phone contact.

All study end points and bleeding events were confirmed based on documents collected from each hospital and were centrally adjudicated by the clinical events committee. Syntax score has been calculated with the application available from www.syntaxscore.com.

**FIGURE 1:**
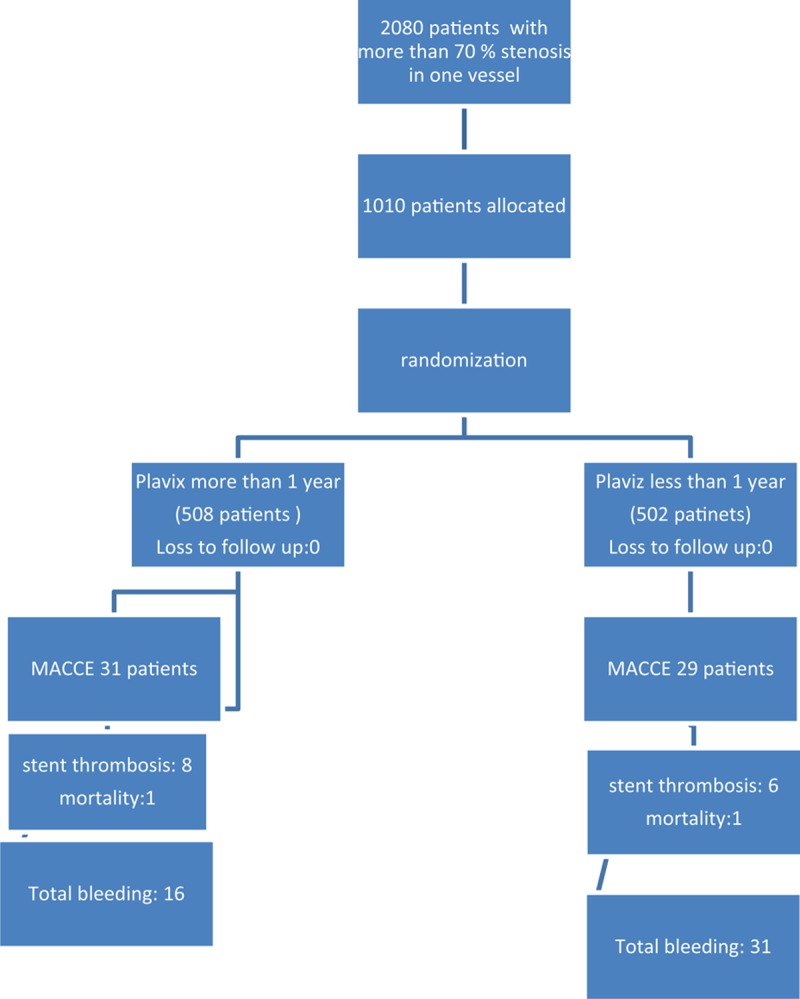
Flow chart of patients enrolled in long-term follow-up on dual antiplatelet therapy. MACCE = major cardiovascular events and bleeding.

The present study was approved by the Ethics Committee of the 2 participating centers independently, participants were provided with verbal information about the study objectives and design; then written informed consent was obtained from them (Figure 1 shows patients allocation).

### Statistical Analysis

Categorical variables are reported as frequency and percentage, whereas continuous variables are reported as the mean ± standard deviation. Kaplan–Meier method was used to estimate the cumulative rate of major adverse cardiovascular events (MACCE), and these mentioned events were compared with the log-rank test. Cox regression analysis was done with interaction testing to show whether the effect of DAT duration on the primary efficacy end point at 2 years was consistent across important prespecified subgroups. A 2-sided *P* value of <0.05 was considered as significant. All analyses were conducted based on the intention-to-treat principle, and were performed using SPSS software version 14.0.

## RESULTS

Totally 1010 patients (363 women and 647 men) participated in this study. They were recruited and randomized to receive DAT more or less than a year; 508 patients were assigned to long-term DAT and 502 patients were assigned to <1 year DAT by simple randomization. Mean age of the participants was 60 ± 10; about 28.0% of the participants had a history of diabetes mellitus. Nearly 54.65% of the participants were presented with hypertension (mean BP of 163.95 range of 145–170), 33.56% were smokers, 2.77% had kidney disorders, 1.28% had lung disorders, and 0.49% had liver disorders. About 2.6% of the participants had autoimmune disorders and 6.8% of them were alcoholics; 5.5% had anemia, 7.2% had peptic ulcer, and 18.0% had gastritis. More than half of the participants had multivessel disorders. The patients’ background history is summarized in Table 1 and the different types of stents which were used are listed in Table 2.

**TABLE 1:**
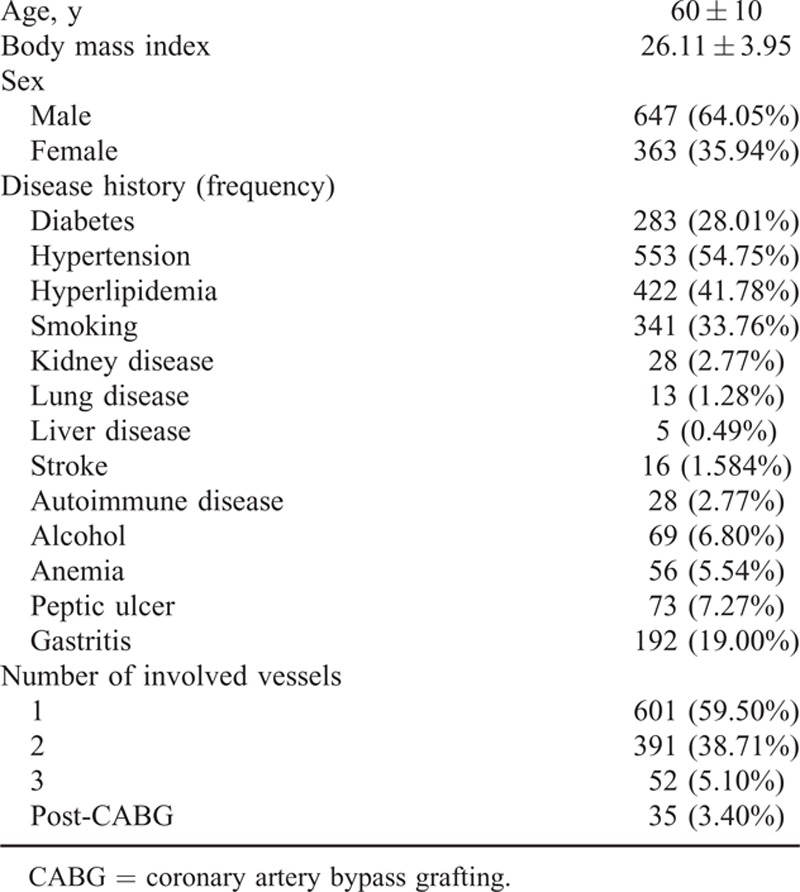
History and Underlying Diseases in Patients Who Underwent Long-Term Dual Antiplatelet Therapy

**TABLE 2:**
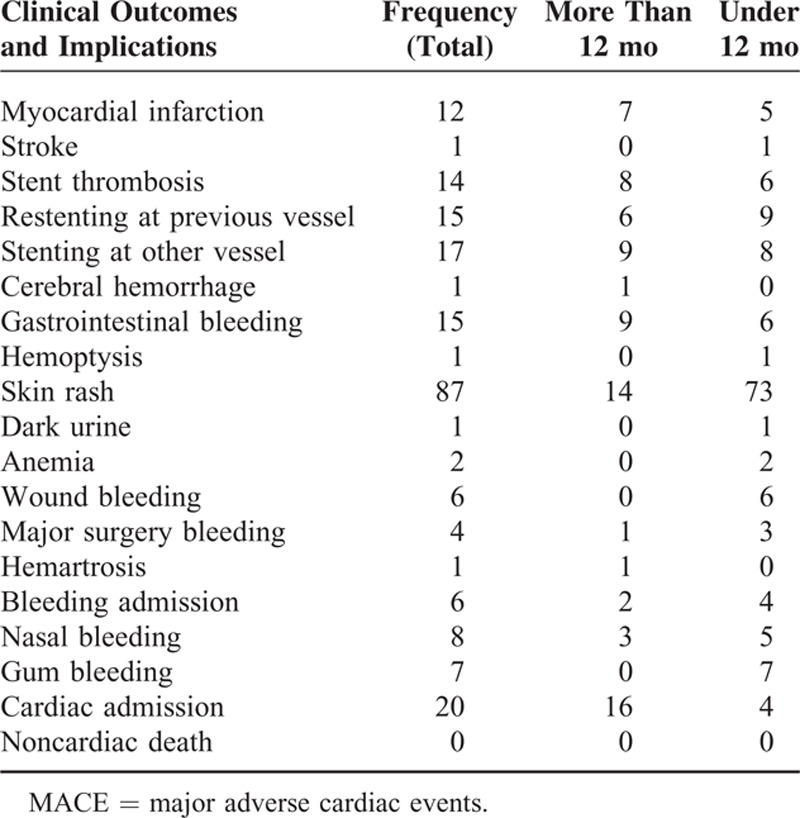
Major and Minor Clinical Outcomes in the Patients Who Underwent Long-Term Dual-Antiplatelet Therapy (MACE)

The most frequent underlying diseases in participants of the present study included hypertension in 54.65% followed by hyperlipidemia in 41.78% and diabetes in 28.01%. The most important nondisease risk factor was smoking in 341 (33.56%) patients (Table 1).

Stenting was performed for small vessels in 217 (21.48%) of participants, at vessel bifurcation sites in 351 (34.75%) of participants, and at an ostial location in 685 (67.82%) of participants. In 136 of patients (13.46%) participated in the present study, overlapping stents occurred.

More than half of the participants (601, 59.50%) who required angioplasty for 1 vessel, 391 (38.71%) for 2 vessels, 52 (5.10%) for 3 vessels, and in 35 (3.40%) patients angioplasty was performed in the grafted vessels after coronary artery bypass grafting (post-CABG). The size of the vessels through which stents were inserted and frequency of involved vessels are demonstrated in Table 3.

**TABLE 3:**
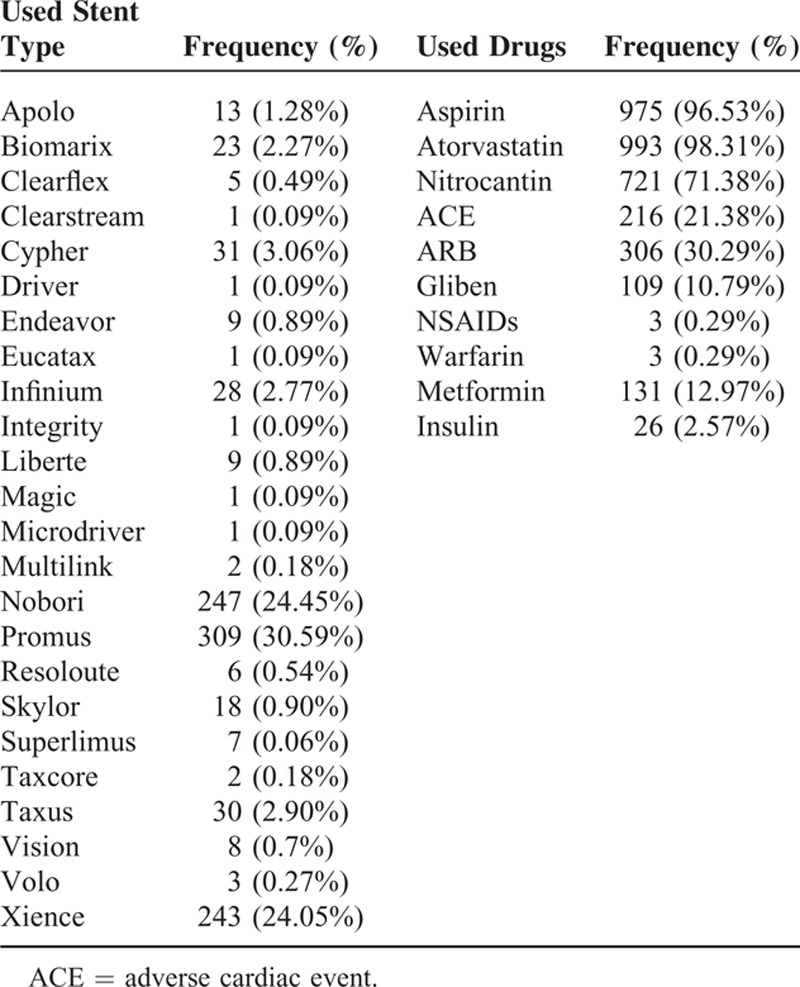
Frequency of Stent Types and Used Drugs in the Patients Who Underwent Long-Term Dual-Antiplatelet Therapy

Table 4 summarizes the clinical outcomes of DAT lasting for a year or less and more than a year. No cases of cardiac death were reported, and solely 2 participants have died of noncardiac causes. In 1 of the participants, cerebral hemorrhage occurred 23 months after stenting was done. A total of 12 cases of myocardial infarction occurred, including 5 cases <12 months after stenting and 7 cases after 12 months. One patient experienced stroke within 12 months of stenting.

**TABLE 4:**
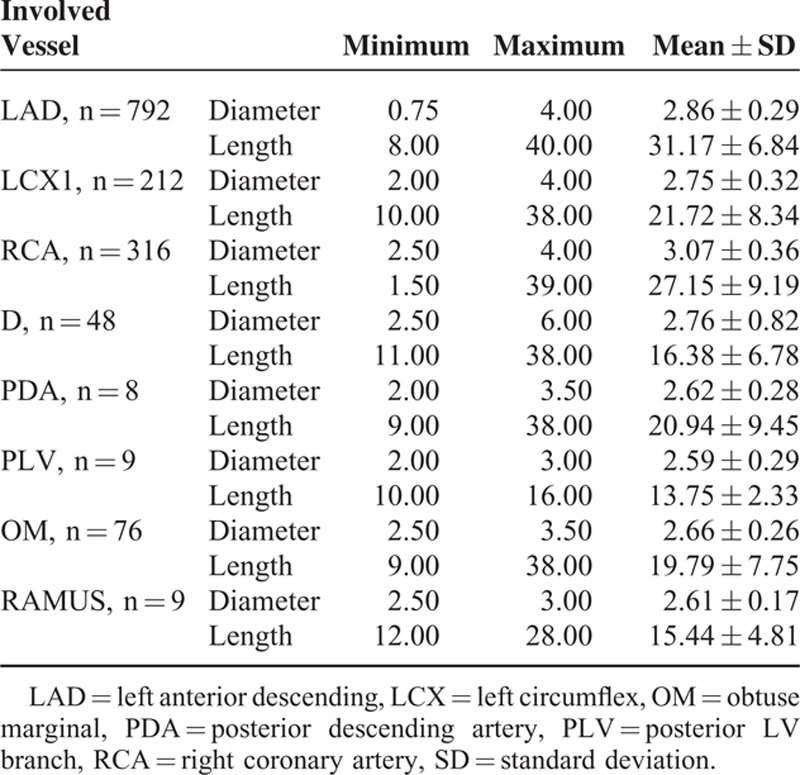
Characteristics of Involved Vessels Needed Stents

Stent thrombosis occurred in 14 patients; in 6 of these cases the thrombosis occurred within 12 months. Restenting was done in 9 of the participants before 12 months and in 6 of them after 12 months. Seventeen patients needed stenting for other new vessels; this was done within 12 months in 8 patients and during the 2nd or 3rd year in 9 patients. One case of cerebral hemorrhage occurred after 12 months. Twenty participants were admitted for cardiac problems were observed; 4 patients within 12 months and 16 patients after 12 months.

Among the 1010 participants in the present study, 1 participant died (0.09%) in the 12-month group and in 1 (0.09%) in the >12-month group. Fifteen patients had stent thrombosis including 6 patients within 12 months, 2 patients during the 2nd year of the study, and 6 patients after 24 months. Overall, restenting was done for 15 (1.48%) patients in previously stented vessels, and stenting in another new vessel was done in 17 (1.63%) patients. Fifteen patients (1.48%) had vessel calcification at the site of stent implementation. All of our patients used Plavix (Sanofi Aventis) during the study. Other drugs used for our patients are listed in Table 2.

Mean score of Syntax was 23.00 ± 5.08. MACCE were recognized in 28 patients. The frequency of AHA class B2 was 21.42% in patients with MACCE and 27.90% in patients without MACCE. Twenty-two (78.57%) of the participants with MACCE and 708 (72.09%) of the participants without MACCE were categorized as AHA class C. Mean score of Syntax was 25.00 ± 5.27 in patients with MACCE, and this score was not significantly different compared to patients without MACCE (*P* = 0.057; Table 5).

**TABLE 5:**
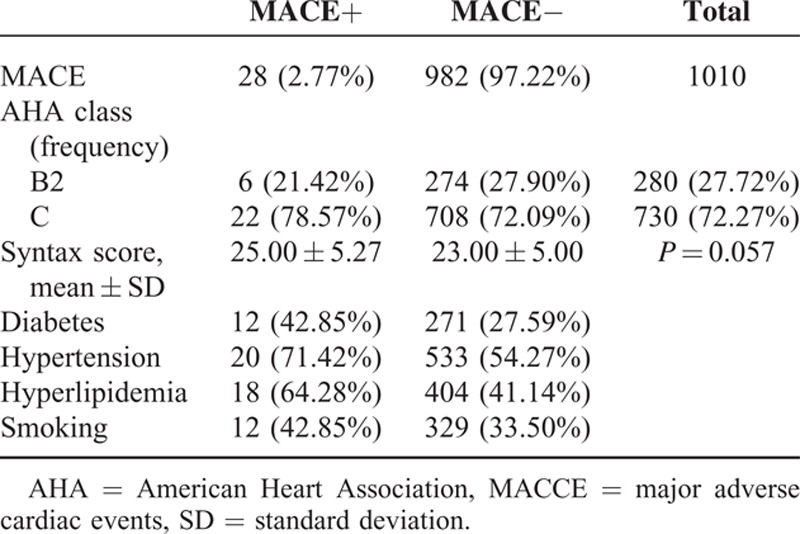
Comparison of AHA Class and Syntax Score in Patients With and Without MACE and Major Risk Factors

## DISCUSSION

Coronary stenting induces an inflammatory response in targeted vessel's intima which causes in stent restenosis. In order to overcome this important issue, drug-eluting stent was invented which decreases inflammatory response and decreases neointimal hyperplasia. This decrease in inflammatory response impairs repair and prohibits natural endothelial coverage of stents, and exposes blood to uncovered stent and increases chance of stent thrombosis and new cardiac events.^7,11^ The time of intimal repair and decrease in chance of stent thrombosis depend on type of stent, active drug, polymers, and other technical and intrinsic factors which were not clearly discovered. This mandates long-term DAT to overcome this disaster, but the optimal duration of DAT still not definitely defined due to vast majority of different results in prior studies.^7,11^

In the present study, as a prospective real life protocol, patients with unstable and stable coronary artery disease were treated by different types of stent, with different polymers and structures, which makes very different population that affects outcomes. There was no significant benefit associated with the continued use of clopidogrel plus aspirin compared to clopidogrel therapy for 1 year, followed by aspirin alone, in reducing the incidence of MACCE. In 1 study, clopidogrel therapy for 2 years resulted in a significant increase in the number of significant bleeding episodes,^17^ which required medical or surgical procedures, or packed-cell transfusion. Other studies demonstrated that 9 to 12 months use of DAT decreases the incidence of MACCE compared to a 1-month regimen after bare-metal stent implantation.^1,2,12^ However, potentially there was a bias in the results of these studies, conducted more than 10 years ago, by differences in the pretreatment regimen between 2 groups. Therefore, their relevance to current practice is unclear. Numerous observational studies^9,10^ showed positive effect of prolong clopidogrel therapy for at least a year after drug-eluting stent implantation; however, other researches failed to confirm their findings.^8,11,18^

Because the benefits of long-term DAT may not depend on type of coronary stents,^1,2^ the more than 2-year clopidogrel use has been examined in different studies. In some studies, ischemic and bleeding events were too infrequent, to be analyzed precisely and give to valid conclusions.^7^ There was a significant difference between the findings of Park et al^7^ and our findings for bleeding which may simply reflect the small number of hemorrhagic events reported in mentioned study.

Cerebrovascular accidents as well as major bleeding events have not been increased in patients who received prolonged dual type of antiplatelet therapy for more than a year. This result is different from the findings of the previous study.^7^ Minor and major bleeding events were not consistently more frequent in our patients who received dual type of antiplatelet therapy for more than a year.

The Syntax score was used to score and grade the coronary anatomy in terms of the number of lesions and their functional impacts, location, and complexity.^19^ Higher scores demonstrated more complex conditions, greater therapeutic challenges and also a worse prognosis in patients who are revascularized by PCIs.^20^ Higher Syntax scores were obtained by patients with a larger number of involved vessels and those who had more clinical outcomes after stent insertion.

The frequency of AHA class C in patients with MACCE was higher than in patients without MACCE, whereas AHA class B2 was more frequent in patients without MACCE. The Syntax score, however, was not significantly higher in patients with MACCE. The frequency of major risk factors in patients with MACCE was much higher than in patients without MACCE. Some studies reported that MACCE were more prevalent in patients with a history of diabetes,^21,22^ smoking,^23^ hypertension, and hyperlipidemia.^24^

Our results are in line with recently published systematic review of effect of DAT for more than 12 months, which demonstrated that DAT for more than 12 months does not decrease MACCE in bare-metal and drug-eluting stents,^25^ another recently published article conducted in over 9000 patients revealed different results.^13^ There are many different articles with different results which were recently published^26–30^ some in favor and some against use of long-term DAT, which may dictates further studies to clarify this important issue. The differences in type of patient enrollment, spectrum of patients’ baseline criteria, type of stent, their polymers, and many other factors should be controlled to reach a common consensus. There are newly innovatory approaches to change polymers and micro RNA therapies which may shed new light to this complex.^31,32^

## CONCLUSION

The present study demonstrated that the long-term use of dual type of antiplatelet therapy for 2 years or more did not significantly decrease the risk of death from any causes or reasons, myocardial infarction, or cerebrovascular events compared to clopidogrel for a year which were solely followed by aspirin. Long-term DAT was not significantly associated with minor or major bleeding events.

### Advantages

This study included a large number of patients and these patients were followed for more than 24 months. In addition, a large number of clinical parameters were evaluated as long as the study has been conducting.

### Limitations

This is a prospective randomized cohort study which enrolled real-world patients with current routine drug-eluting stents and had the same drawback of prior studies which included different types of stents and, however, this is added to prior literature the safety of long-term DAT, it mandates further studied to compare different types of stent and polymer with sophisticated evaluation of re-endothelialization. No placebo therapy or similar drug was used instead of clopidogrel so the effects of different pharmacological treatments could not be compared.
